# [^68^Ga]FAPI-PET/CT for radiation therapy planning in biliary tract, pancreatic ductal adeno-, and adenoidcystic carcinomas

**DOI:** 10.1038/s41598-022-20447-6

**Published:** 2022-09-28

**Authors:** Nika Guberina, Lukas Kessler, Christoph Pöttgen, Maja Guberina, Martin Metzenmacher, Ken Herrmann, Maja Mucha, Christoph Rischpler, Frank Indenkämpen, Jens T. Siveke, Jürgen Treckmann, Lale Umutlu, Stefan Kasper, Wolfgang P. Fendler, Martin Stuschke

**Affiliations:** 1 Department of Radiotherapy, West German Cancer Center (WTZ), University of Duisburg-Essen, University Hospital Essen, Hufelandstraße 55, 45147 Essen, Germany; 2 Department of Nuclear Medicine, University of Duisburg-Essen and German Cancer Consortium (DKTK)-University Hospital Essen, Essen, Germany; 3 Department of Medical Oncology, West German Cancer Center, University Hospital Essen, Essen, Germany; 4 German Cancer Consortium (DKTK), Partner Site University Hospital Essen, Essen, Germany; 5 Division of Solid Tumor Translational Oncology, German Cancer Consortium (DKTK), Partner Site University Hospital Essen, and German Cancer Research Center (DKFZ), Heidelberg, Germany; 6 Bridge Institute of Experimental Tumor Therapy, West German Cancer Center, University Hospital Essen, Essen, Germany; 7 Department of General, Visceral and Transplantation Surgery, University of Duisburg-Essen, University Hospital Essen, Essen, Germany; 8 Institute of Diagnostic and Interventional Radiology and Neuroradiology, University of Duisburg-Essen, University Hospital Essen, Essen, Germany

**Keywords:** Cancer, Anatomy, Medical research, Oncology

## Abstract

Biliary-tract-carcinomas (BTC), pancreatic-ductal-adenocarcinomas (PDAC) and adenoidcystic-carcinomas (AC) have in common that they are traditionally treated with large clinical-target-volumes (CTV). The aim of this study is to examine the impact of pretreatment-[^68^Ga]FAPI-PET/CT on target-volume-definition and posttreatment-[^68^Ga]FAPI-PET/CT-response-assessment for BTC-, PDAC- and AC-patients referred to radiation-therapy. All consecutive BTC-, PDAC-, and AC-patients who received pretreatment-[^68^Ga]FAPI-PET/CT±[^18^F]FDG-PET/CT were included from 01.01.2020 to 01.03.2022. MTV and SUV_max_ were separately generated based on [^68^Ga]FAPI- and [^18^F]FDG-PET/CT-images. A [^68^Ga]FAPI- and [^18^F]FDG-based-CTV was defined. Treatment-plans were compared. Treatment-response was reassessed by a second [^68^Ga]FAPI-PET/CT and [^18^F]FDG-PET/CT after treatment-completion. Intermodality comparison of lesion-to-background-ratios [SUV_max_lesion_/SUV_mean_background_] for individual timepoints *t*_*1*_ and *t*_*2*_ revealed significant higher values for [^68^Ga]FAPI compared to [^18^F]FDG (*t*_*1*_, *p* = 0.008; *t*_*2*_, *p* = 0.005). Intermodality comparison of radiation-therapy-plans showed that [^68^Ga]FAPI-based planning resulted in D100% = 97.2% and V95% = 98.8% for the [^18^F]FDG-MTV. [^18^F]FDG-based-planning resulted in D100% = 35.9% and V95% = 78.1% for [^68^Ga]FAPI-MTV. [^18^F]FDG-based-planning resulted only in 2 patients in V95% > 95% for [^68^Ga]FAPI-MTV, and in 1 patient in D100% > 97% for [^68^Ga]FAPI-MTV. GTV-coverage in terms of V95% was 76.4% by [^18^F]FDG-based-planning and 99.5% by [^68^Ga]FAPI-based-planning. Pretreatment [^68^Ga]FAPI-PET/CT enhances radiation-treatment-planning in this particular group of patients. While perilesional and tumoral follow-up [^18^F]FDG-uptake behaved uniformly, perilesional and tumoral reaction may differ in follow-up [^68^Ga]FAPI-imaging. Complementary [^68^Ga]FAPI- and [^18^F]FDG-imaging enhance treatment-response-assessment.

## Introduction

For curative-intent radiotherapy high-end multimodal imaging is crucial for precision therapy. High-end imaging allows delivering highly conformal doses to the tumor by, at the same time, minimizing radiation dose to healthy tissue. Nowadays, target volume delineation based on PET/CT-images has become reference standard in radiotherapy planning for many tumor entities, entering into national and international guidelines from brain, thoracic to pelvic malignancies. In a prospective multicentre trial, 2-deoxy-2-[^18^F]fluoro-d-glucose ([^18^F]FDG) [^18^F]FDG-PET/CT altered treatment management in ~ 72% of patients with lung cancer, in 85% of patients with pancreatic ductal adenocarcinomas and 78% of patients with cancer of unknown origin^1^. The mean adaptation rate of radiotherapy treatment plans based on [^18^F]FDG-PET was 55.4% (range 44.0–69.2%), with major treatment plan modifications made in 43.3–68.2% of cases^1^. Typically the gross tumor volume (GTV) comprises PET-positive lesions that will be expanded to the clinical target volume (CTV) by 5–8 mm margins with respect of anatomic boundaries^2,3^. Complementary functional and morphological changes allow an accurate non-invasive assessment of disease spread and activity. Multimodality staging by EBUS-TBNA and PET-imaging allowed the identification of frequent patterns of lymphogenic metastatic spread in non-small lung cancer^4^. Furthermore, early phase-II-trials were conducted to examine the value of interim [^18^F]FDG-PET/CT-imaging for delineation of residual metabolic target volumes in patients with locally advanced non-small cell lung cancer^5,6^. These randomized trials were promising, allowing for improved local tumor control by focused radiation dose escalation targeted to the residual [^18^F]FDG-avid tumor^5,6^. However, the corresponding subsequent randomized trial yielded no confirmatory results^7^.

Metabolic shrinkage may be greater than plain anatomic changes^5^. Thus, it can represent a valid prognostic parameter of interim [^18^F]FDG-PET/CT-imaging as supported by a large retrospective study examining post-induction chemotherapy, interim PET-parameters^8^. Interim [^18^F]FDG-PET/CT-imaging is reported to have a significant progonstic impact and to represent a valuable tool for guiding individualized treatment intensification^8^.

For a long time [^18^F]FDG-PET/CT was the most widely used application of molecular imaging for initial tumor staging and restaging. However, now, more and more studies predict the end of the hegemony of [^18^F]FDG-PET/CT^9^. Randomized prospective, phase-III trials have been designed to determine, whether PSMA-PET/CT-imaging can improve outcomes in patients with prostate cancer^10^, combined [^18^F]FDG- and [^68^Ga]DOTATATE PET/CT for noninvasive assessment of tumor heterogeneity in G2 and G3 NETs^11^, MET-PET reported a useful modality for the diagnosis of radiation-induced changes in brain metastases with SUV_max_^12^ or MET-PET and random-forest based analysis for outcome prediction of patients with brain tumors and for differentiating recurrent brain tumor from radiation necrosis^13^ are only a few of a whole series of studies building the architecture of diagnostic prospects.

Recent developments bring the fibroblast activation protein (FAP) inhibitor (FAPI) as PET tracer in oncology forward. [^68^Ga]FAPI represents a novel radiopharmacon that may be used as diagnostic or therapeutic target. The FAP-specific enzyme inhibitor (FAPI) binds to FAP by blocking its chemical reaction. FAP represents a transmembrane glycoprotein expressed on cancer-associated fibroblasts (CAFs) of several tumor entities^14^. These non-neoplastic cells modulate the tumor microenvironment and the extracellular matrix^15^. They are reported to modify tumor progression and therapeutic response. [^68^Ga]FAPI is reported to concentrate in tumours at a high proportion, whereas it is quickly hived off in normal tissue^16^ and organs at risk. Particularly, in tumor entities of the head and neck, which are in close vicinity to the nasal sinus, Waldeyer's tonsillar ring or in the region of the upper abdomen such as close to the liver and biliary tract, viz. close to normal tissues with a high physiological [^18^F]FDG-uptake, target delineation may be challenging^17,18^. At the same time biliary-tract-carcinomas (BTC), pancreatic-ductal-adenocarcinomas (PDAC) and adenoidcystic-carcinomas (AC) have in common that they are traditionally treated with large clinical-target-volumes. There exists a strong need for a better tumor, and at the same time better target delineation on imaging. Therefore, the present study focusses on the value of [^68^Ga]FAPI for target delineation and radiation therapy planning of adenoidcystic carcinomas, pancreatic ductal adenocarcinomas (PDAC), and biliary tract cancer.

In adenoidcystic carcinomas, because of the spread along the nerve sheaths, large CTV safety margins are used in radiotherapy to prevent local recurrence. A more sensitive detection of tumor spread along these routes is desirable to individualize, reduce the target volumes and thus allow a higher dose^19^. In a study of 12 patients with adenoidcystic carcinoma the clinical potential for staging and radiotherapy planning was assessed by means of [^68^Ga]FAPI-PET/CT^20^. The authors conclude that [^68^Ga]FAPI represents a promising imaging modality for adenoidcystic carcinoma, improving the precision of staging and radiotherapy planning compared to CT and MRI^20^.

For pancreatic ductal adenocarcinomas (PDAC) conventionally fractionated radiotherapy with concurrent chemotherapy using elective nodal irradiation either in the neoadjuvant or adjuvant setting was largely unsuccessful in terms of improving survival^21,22^. Stereotactic radiotherapy is evolving for locally advanced PDAC, but with small margins a precise determination of tumor spread is mandatory^23,24^. For biliary tract cancer only phase-II-trials are available, that used large target volumes^25^.

The SUV_max_ of [^18^F]FDG PET/CT is reported to aid differential diagnosis of solitary pancreatic lesions and also in the prediction of proliferative activity of pancreatic cancer^26^. However, while for many malignant tumors [^18^F]FDG-uptake correlated with cancer proliferation, for PDAC this correlation remains controversial^27^. According to a study examining the metabolic landscape of patients with advanced biliary tract cancer lesions, a high SUV_max_ uptake was associated with a poorer differentiated histology than of those with a low SUV_max_ uptake^28^. Likewise, the authors identified SUV_max_ as a prognostic factor for overall and progression-free survival in biliary tract cancer^28^. Quite the reverse, accurate delineation of lesions with a low or moderate proliferation may be difficult due to a poor [^18^F]FDG-uptake.

The aim of the present study is to examine the impact of pre- and posttreatment [^68^Ga]FAPI-PET/CT on radiotherapy target volume definition and response assessment for patients with adenoidcystic carcinoma, PDAC and biliary tract cancer referred to radiation therapy. Furthermore, the role of [^68^Ga]FAPI-PET/CT and [^18^F]FDG-PET/CT in radiation treatment shall be identified and discussed in this specific, for imaging and precise treatment planning challenging, tumor entities.

## Methods

### Patient cohort

**Figure 1:**
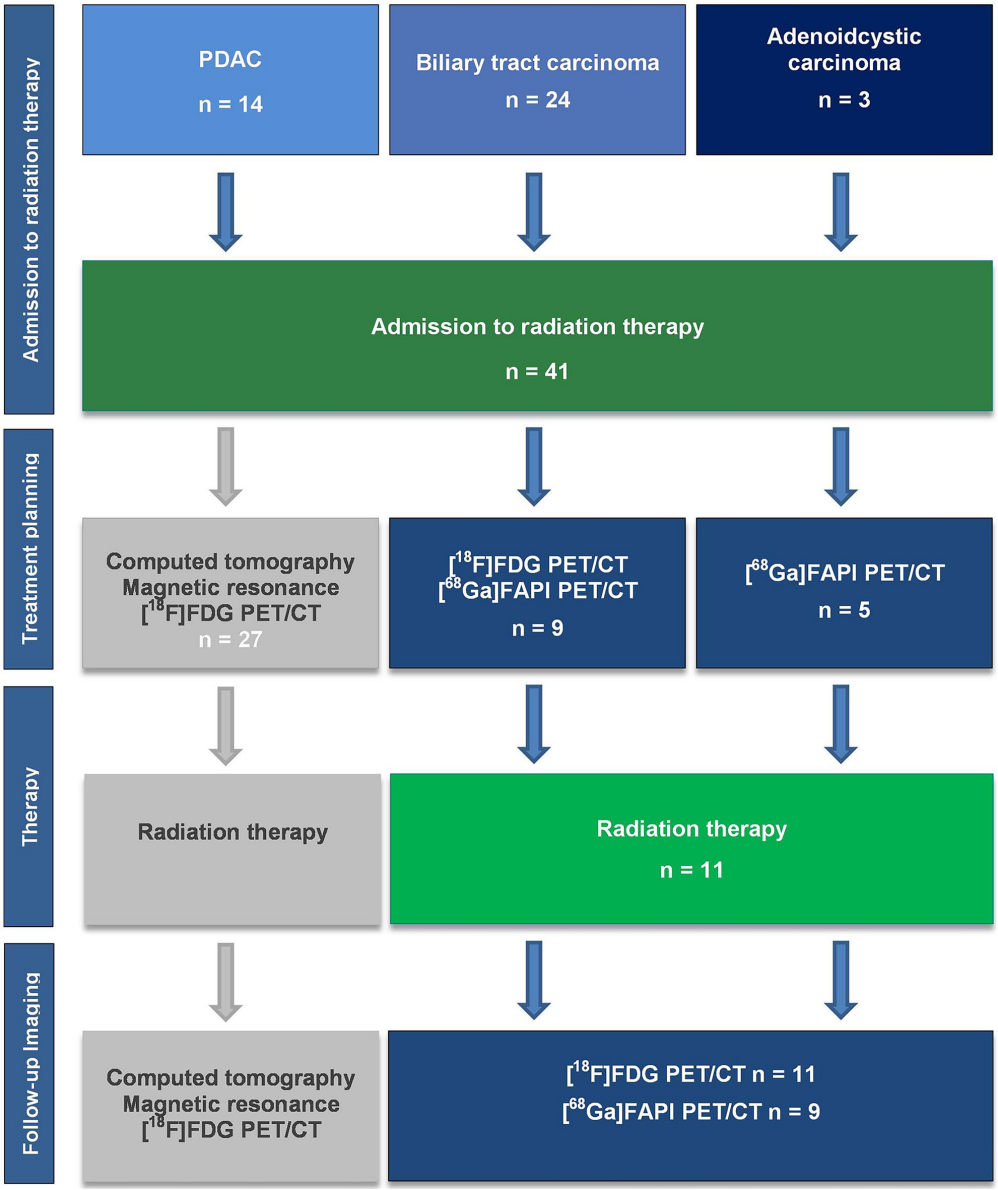
Consort diagram: all consecutive patients with pancreatic ductal adenocarcinoma (PDAC), biliary tract and adenoidcystic carcinoma were reviewed in the time period from 01.01.2020 to 01.03.2022. For study examination those patients who received a [^68^Ga]FAPI-PET/CT prior to radiation therapy admission were included.

All consecutive patients with PDAC, biliary tract and adenoidcystic carcinoma who were introduced to the department of radiation therapy were reviewed for study purposes in the time period from 01.01.2020 to 01.10.2021. For study examination those patients who received a [^68^Ga]FAPI-PET/CT prior to radiation therapy admission were included (Consort diagram Fig. 1). [^68^Ga]FAPI-PET/CT was performed for clinical purposes such as high risk situations, challenging tumor delineation on conventional imaging and the necessity for assessment of complete tumor extension prior to radiation therapy. Patients were enrolled in a prospective observational trial conducted at University Hospital Essen (NCT04571086), which was approved by the local ethics committee of University Hospital Essen (19-8991-BO). Patients gave written informed consent for inclusion. Patients gave written informed consent for anonymized analysis of the data. All research was performed in accordance with relevant guidelines/regulations and in accordance with the Declaration of Helsinki.

### Pretreatment imaging and data processing

All included patients were introduced to the Department of Radiation Therapy and received a pretreatment [^68^Ga]FAPI-PET/CT prior to or at time of admission to radiation therapy. Some patients underwent sequential [^68^Ga]FAPI-PET/CT and [^18^F]FDG-PET/CT on the same day or within a narrow time interval prior to treatment planning after patient consent. All examinations were performed on the PET/CT scanner Biograph mCT or Biograph mCT VISION (Siemens Healthineers, Erlangen, Germany). PET acquisition and reconstruction was completed by ordered subset expectation maximization (OSEM) based algorithms. For post-reconstruction filtering a Gaussian filter kernel with a full width at half maximum of 4 mm was applied. Data collected on the PET/CT system were reconstructed with a pixel spacing of 1.65 × 1.65 × 3 and rows and columns of 400 × 400. PET image reconstructions were performed by modeling of resolution degrading phenomena, viz. point-spread function (PSF) reconstruction. Attenuation correction was achieved either by low dose or contrast enhanced, diagnostic computed tomography. Furthermore, the diagnostic CT-component served for morphological correlation. All patients who in interdisciplinary consensus and after radiation therapy specialist assessment were scheduled for radiation therapy underwent a treatment planning simulation. Patients received a planning CT of the area being treated with or without contrast-agent, iterative reconstruction and automatic dose modulation.

### Lesion and background definition

The bio-distribution of [^68^Ga]FAPI- and [^18^F]FDG was determined in terms of metabolic tumor volumes (MTV) and maximum standardized uptake values (SUV_max_). SUV_max_ was determined by a region grow algorithm for the index lesion. Time interval between examinations of patients who received both, a [^68^Ga]FAPI- and [^18^F]FDG-examination, was in mean 5.3 days (median 0 days, range 0–35 days) without treatment changes in-between. In a second step the MTV and SUV_max_ were separately generated on the basis of [^68^Ga]FAPI- and [^18^F]FDG-PET/CT-images. By means of the SUV_max_ thresholding tool implemented in the treatment planning software ECLIPSE with a standardized threshold of 40% and supervised adaptation the index lesion was measured with respect to the planned radiation treatment.

**Figure 2:**
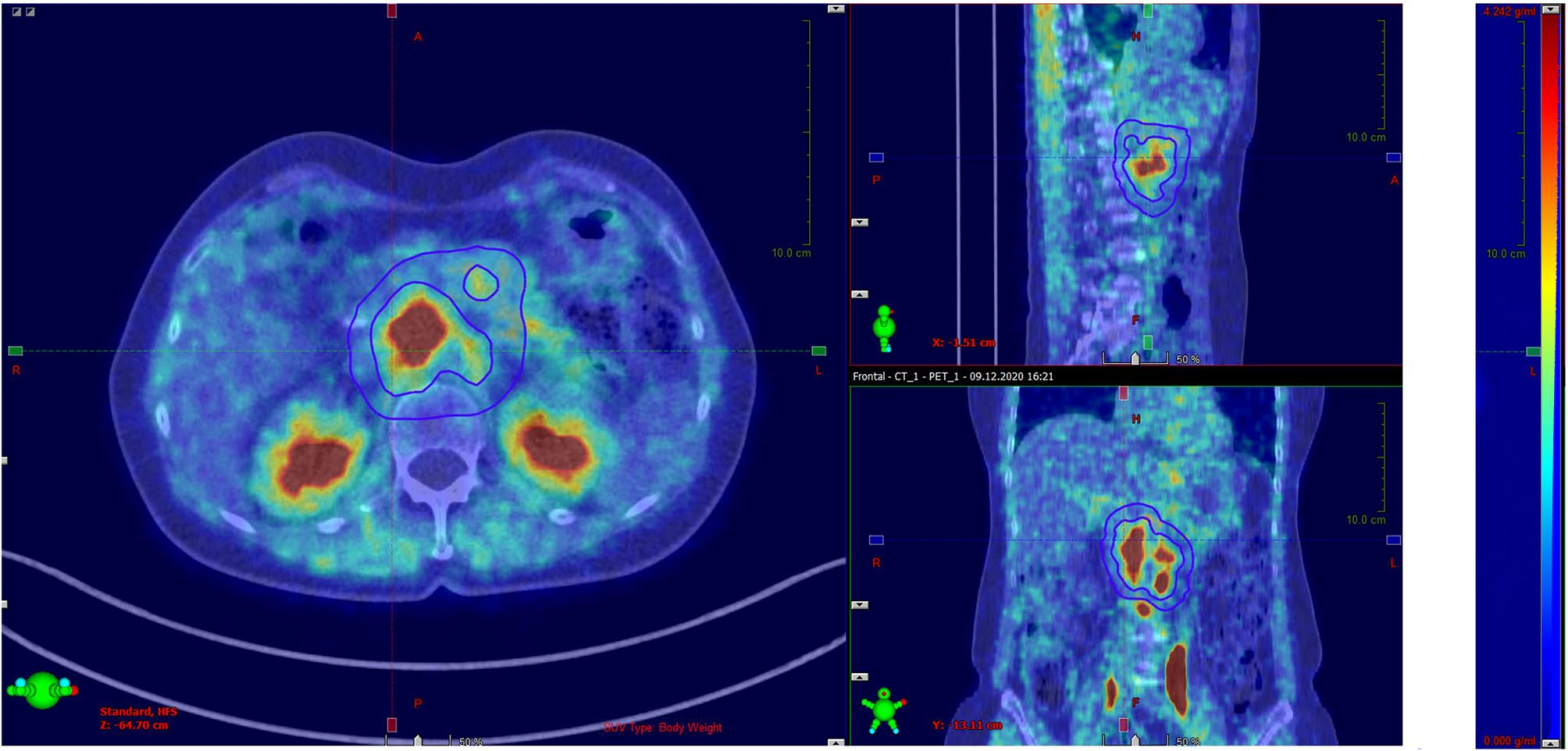
Showing patient H with a lymphonodular metastatic pancreatic ductal adenocarcinoma at timepoint *t*_*1*_. Fused mode [^68^Ga]FAPI-PET/CT displaying assessment of physiological tracer activity distribution and the surrounding perilesional background uptake examined, left-side: axial reconstruction at the level of the kidneys, upper right-side: midline sagittal reconstruction, lower right-side coronal reconstruction [point-spread function (PSF) reconstruction; PET Rainbow 2D with colour blending yellow-blue, 27 min after 106 MBq [^68^Ga]FAPI-injection]; likewise background uptake was determined (blue structure) for all index lesions in terms of SUV_mean_ in a hollow sphere in 0.5–1.5 cm perilesional distance.

Physiological tracer activity distribution and the surrounding background uptake was examined for all index lesions by determining SUV_max_ and SUV_mean_ in a hollow sphere in 5–15 mm perilesional distance (Fig. 2).

Additionally, to consider the background activity concentration in the segmentation of the MTV_*Background*_ the respective threshold was calculated as described previously^29^:

$$SUVmaxBackground+0.4 \left(SUVmaxTumor-SUVmaxBackground\right)$$

whereby SUV_maxTB_ = SUV_maxTumor_ − SUV_maxBackground._

### Treatment planning

All available pretreatment PET/CT-images were imported to the treatment planning system ECLIPSE (Varian, Palo Alto, USA). Each [^68^Ga]FAPI- and [^18^F]FDG-PET/CT study was reviewed prior to target definition and fused with the standardized treatment planning CT by means of the treatment planning system ECLIPSE. Rigid and deformable image registration was used to fuse the planning CT with the pretreatment [^68^Ga]FAPI- and/or [^18^F]FDG PET/CT. The gross tumor volume (GTV) was defined on the planning CT. The clinical target volume (CTV) was delineated according to respective guidelines in order to encompass possible microscopic tumor extension and to consider residual deformations. In general, a CTV expansion of 5 mm or up to a nearer anatomic border was conducted. The planning target volume (PTV) was determined with 5 mm around the CTV to take potential set-up errors into account. Moreover, to control normal tissue complications all organs at risks were defined on the planning CT with restriction of hot spots outside sensitive areas. A separate clinical [^68^Ga]FAPI- and additional [^18^F]FDG-based PTV was defined. For all except of one patient the [^18^F]FDG-based PTV was generated by a 10-mm margin around the MTV with regard to organs at risk and used to develop the 3-dimensional conformal VMAT radiation therapy plan. In one patient who underwent stereotactic lymph node treatment the [^18^F]FDG-based PTV was generated by a 5-mm margin around the MTV with regard to organs at risk. The two treatment plans for each patient were compared with respect to target volume coverage (GTV, [^68^Ga]FAPI- and [^18^F]FDG-MTV).

For tumors in the upper abdomen radiation therapy was planned in plain free breathing delivered during the exhalation phase with gating-windows of 50% of the breathing cycle. By means of a Respiratory Gating System device (RPM) the exact respiration phase was monitored. The minimum dose to the 98% of the target volumes containing the ordered highest dose bins was required to be as at least 95% of the prescribed dose. The Acuros XB algorithm was used to generate either volumetric modulated arc therapy (VMAT) or static-field intensity modulated radiotherapy (IMRT) plans. Patients received radiation therapy once daily with a fractionation dose of 5 × 1.8–3 Gy/w (q.e.d.) at the linear accelerator TrueBeam (Varian, Palo Alto, USA). In case of stereotactic radiation therapy higher fraction doses were applied (3.5 Gy/F). Daily image guidance (IGRT) was an integral part of every radiation therapy sequence and ensured a precise treatment delivery. Online target matching was accomplished with the help of the 6-degrees of freedom table available in the linear accelerator TrueBeam (Varian, Palo Alto, USA).

### Post-radiation imaging

**Figure 3:**
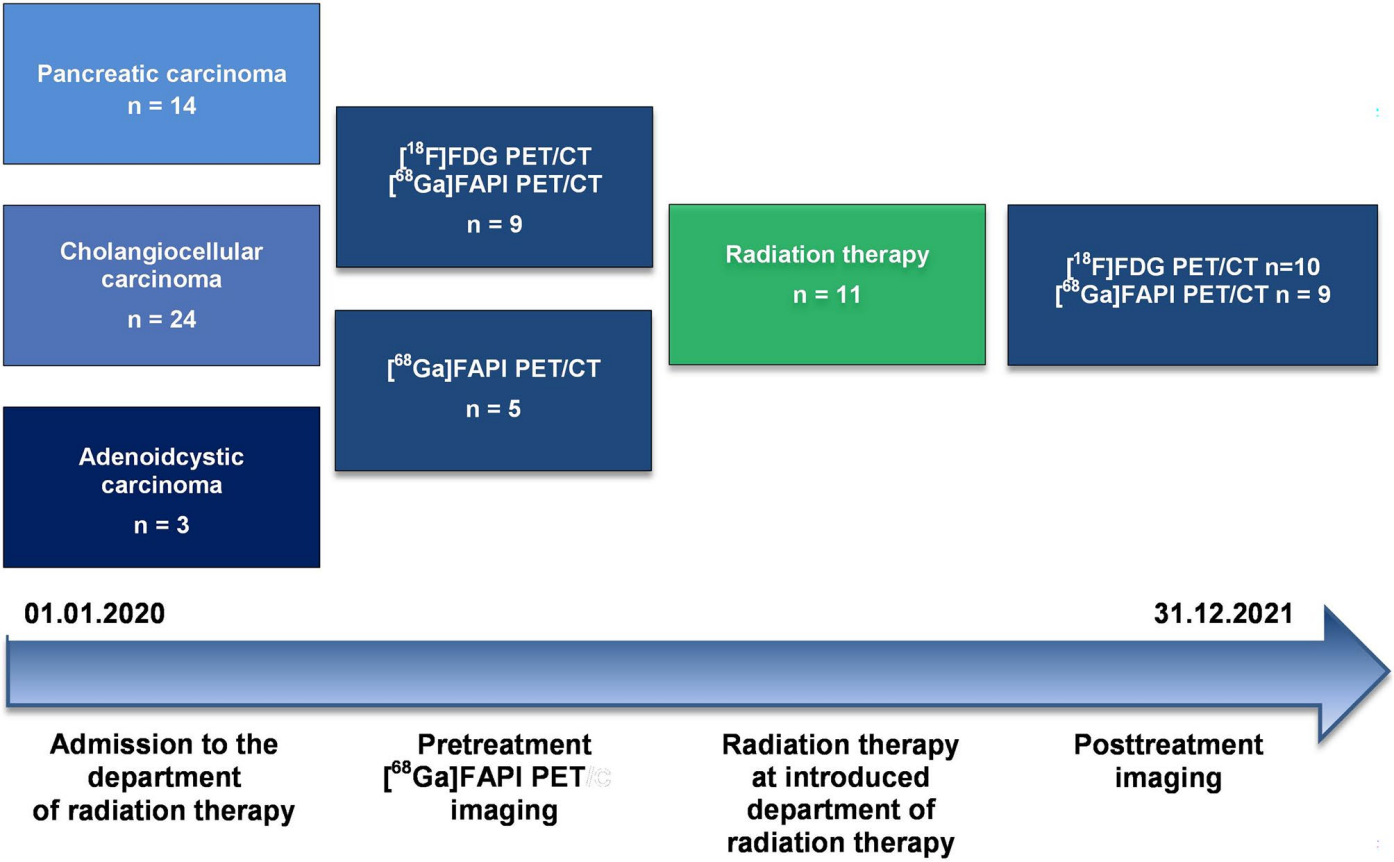
Diagnostic and treatment timeline: all consecutive patients with pancreatic ductal adenocarcinoma (PDAC), biliary tract and adenoidcystic carcinoma were reviewed for study purposes in the time period from 01.01.2020 to 01.10.2021. For study examination those patients who received a [^68^Ga]FAPI-PET/CT prior to radiation therapy admission were included (n = 14).

Treatment response was reassessed in terms of lesion-to-background ratios [SUV_*maxTumor*_/SUV_*meanBackground*_] by the second and/or third [^68^Ga]FAPI-PET/CT and [^18^F]FDG-PET/CT at timepoints *t*_*2*_ and *t*_*3*_ following *t*_*1*_ after treatment completion where available (Diagnostic and treatment timeline highlighted by Fig. 3). Absolute ΔSUV_*maxTumor*_ and ΔSUV_*maxBackground*_ were examined in dependence of time from radiation therapy start to follow-up imaging. Additionally, to evaluate the impact of the response of perilesional background the background reaction was determined in terms of absolute and percentage lesion-to-background difference ΔSUV_maxTB_ [SUV_*maxTumor*_ − SUV_*maxBackground*_] and %ΔSUV_maxTB_ [%SUV_*maxTumor*_ − %SUV_*maxBackground*_] at timepoints *t*_*2*_ and *t*_*3*_ following *t*_*1*_ after treatment.

### Statistical analysis, primary and secondary endpoints

The primary endpoint is to examine the impact of [^68^Ga]FAPI-PET/CT diagnostics on target volume definition prior to radiation therapy and coverage of morphological GTV by [^68^Ga]FAPI- and [^18^F]FDG-PTV based planning. Secondary endpoints represent the assessment of intermodality differences between [^68^Ga]FAPI- and [^18^F]FDG PET/CT for target volume definition, for radiation therapy planning and for assessment of post-radiation treatment response.

Intermodality comparison between [^68^Ga]FAPI- and [^18^F]FDG-PTV based planning was examined in terms of D_100%_, MTV, as well as perilesional SUV_max_, SUV_mean_ and lesion-to-background ratios [SUV_max_lesion_/SUV_mean_background_] for individual timepoints *t*_*1*_ and *t*_*2*_ by using non-parametric paired *t*-tests (Wilcoxon signed rank test). Kruskal Wallis test was applied to examine lesion-to-background ratios of the three tumor entities. To test for consistent differences between the percentage change of [^68^Ga]FAPI- and [^18^F]FDG-uptake from timepoint *t*_*1*_ to *t*_*2*_ the sign-test was used. All descriptive and statistical analyses were performed using IBM SPSS Statistics version 27 (IBM Corp., Armonk, New York, USA).

## Results

Altogether 41 patients with PDAC (n = 14), biliary tract (n = 24) and adenoidcystic carcinoma (n = 3) were introduced to the department of radiation therapy in the time period from 01.01.2020 to 01.03.2022 (Fig. 1). In total, 14/41 patients (34.1%) received a [^68^Ga]FAPI-PET/CT-examination for clinical purposes prior to radiation therapy after patient consent, 9/41 patients (21.9%) underwent a [^18^F]FDG PET/CT on the same day or within a narrow time interval prior to treatment planning. Median time of [^68^Ga]FAPI-PET/CT prior to radiation therapy initiation was 34 days (mean 28.9, range 4–78 days). Overall 11/14 patients who received a [^68^Ga]FAPI-PET/CT-examination underwent a radiation therapy at the department of radiation therapy introduced. One patient underwent radiation therapy at a different site with carbon ions instead of photons. 2/14 patients underwent no radiation therapy due to different reasons, one patient due to newly diagnosed peritoneal carcinomatosis, the other due to a secondary re-resection with sufficient surgical margins. 9/11 patients who underwent radiation therapy, and 10/14 considering all patients, received a 2nd [^68^Ga]FAPI-PET/CT. 10/11 patients who underwent radiation therapy, and 11/14 patients considering all patients referred to the Department of Radiation Therapy, received a follow-up [^18^F]FDG-examination (Fig. 1). 2 patients underwent a 3rd [^68^Ga]FAPI-PET/CT and 3 patients a 3rd follow-up [^18^F]FDG-examination. The median activity of the pretreatment [^68^Ga]FAPI-PET/CT was 95 MBq (mean injected 107 MBq, range 52–177) with images acquired in mean 21 min (range 10–61 min) post-injection. The median activity of the pretreatment [^18^F]FDG was 307 MBq (mean injected 286 MBq, range 101–431), with images acquired in mean 67 min (range 51–91 min) post injection. The median activity of the follow-up [^68^Ga]FAPI-PET/CT was 94 MBq (mean injected 92 MBq, range 80–101) with images acquired in mean 28 min (range 10–68 min) post-injection. The median activity of the follow-up [^18^F]FDG was 289 MBq. (mean injected 297 MBq, range 227–440), with images acquired in mean 71 min (range 54–90 min) post injection. Table 1 summarizes patient characteristics A–N (Table 1 and Supplementary Table 1).

**Table 1:** Patient characteristics of all consecutive patients with pancreatic ductal adenocarcinomas (PDAC), cholangiocellular/biliary tract (BTC) and adenoidcystic carcinoma (total n = 14) receiving [^68^Ga]FAPI-PET/CT for radiation therapy planning (entity; RTx: conducted RTx 1 yes, 0 no; time interval to RTx-start in days; Fx (fractionation dose in Gy); and total dose (in Gy)) [adenoidcystic carcinoma A–C, biliary tract carcinoma D–G, PDAC H–N].

Patient ID	Entity	RTx	Time interval RTx-start (days)
A	Adenoidcystic carcinoma	0	
B	Adenoidcystic carcinoma	1	6.0
C	Adenoidcystic carcinoma	0	
D	BTC	1	18.0
E	BTC	1	34.0
F	BTC	1	78.0
G	BTC	1	7.0
H	PDAC	1	34.0
I	PDAC	0	
J	PDAC	1	4.0
K	PDAC	1	35.0
L	PDAC	1	34.0
M	PDAC	1	6.0
N	PDAC	1	55.0

**Figure 4:**
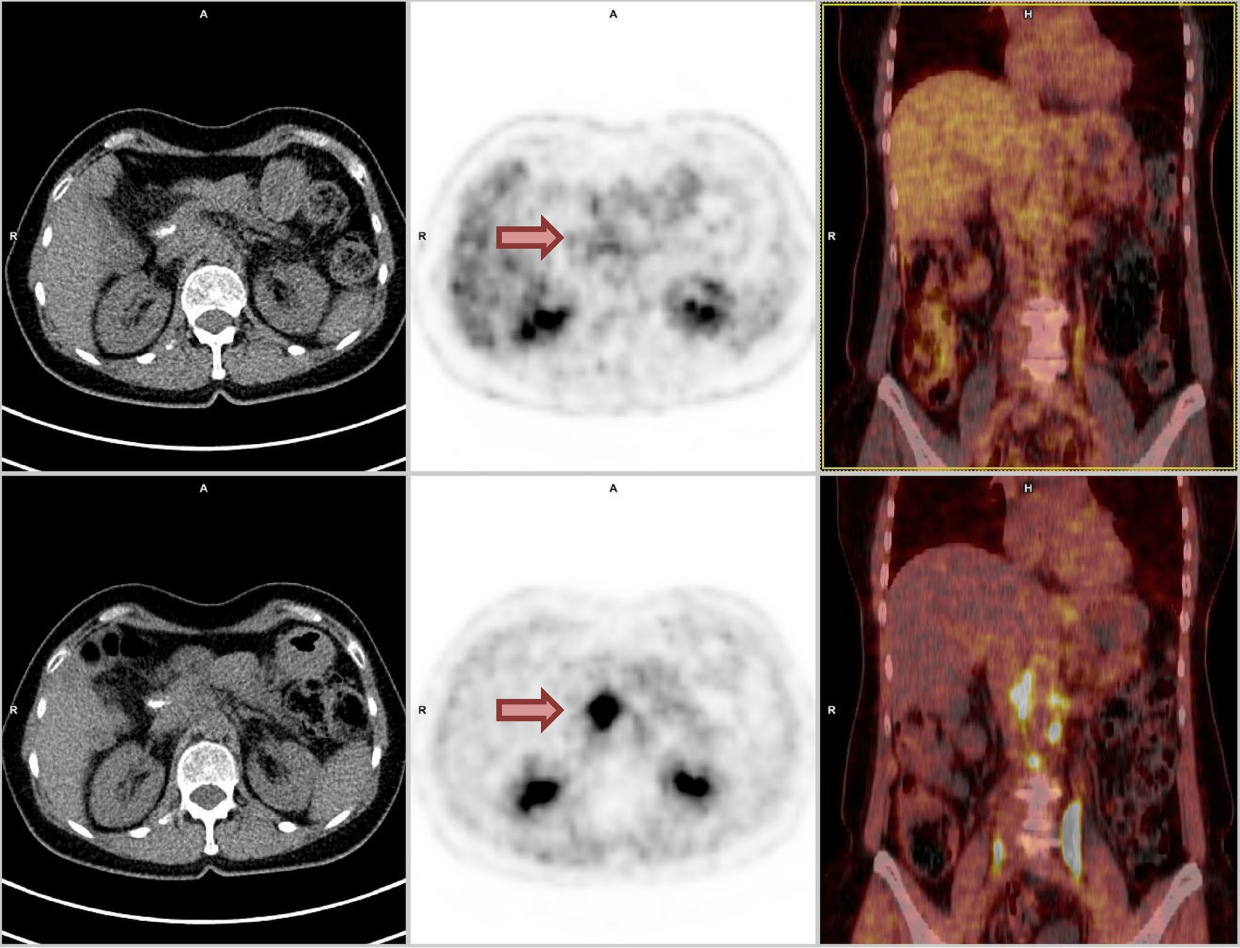
Showing patient H with a lymphonodular metastatic pancreatic ductal adenocarcinoma at timepoint *t*_*1*_. Upper and lower left-side: unenhanced CT; upper middle column: [^18^F]FDG-PET; lower middle column: [^68^Ga]FAPI-PET (both axial reconstructions at the level of the kidneys); upper and lower right-side: fused mode [^18^F]FDG-PET/CT (upper row) and [^68^Ga]FAPI-PET/CT (lower row) displaying assessment of index lesions (red arrows) [point-spread function (PSF) reconstruction; PET Heat 2D, 55 min after 101 MBq [^18^F]FDG injection and 27 min after 106 MBq [^68^Ga]FAPI-injection]. Uptake on [^18^F]FDG-PET-images [^18^F]FDG-uptake was minimal and only slightly elevated compared to background noise.

The [^68^Ga]FAPI-PET/CT-findings altered the radiation therapy volume in 7/8 examined patients (87.5%) in comparison to the [^18^F]FDG-PET MTV, considering the 8 patients de facto treated at this institution’s department of radiation therapy, who received both, an initial [^18^F]FDG-PET and [^68^Ga]FAPI-PET for treatment planning. With regard to the MTV as the basis for treatment planning, [^68^Ga]FAPI-based MTV was significantly larger compared to [^18^F]FDG-based MTV (*p* = 0.008). Tumor lesions were detected by [^68^Ga]FAPI-PET in all 14 patients. On the contrary, on [^18^F]FDG-PET in 2 patient with histopathologically confirmed PDAC [^18^F]FDG-uptake was minimal and only slightly elevated compared to background noise (Fig. 4).

[^68^Ga]FAPI-PET detected two additional patients with suspected liver metastases, whereas one turned out to be unspecific tracer-uptake in the follow-up imaging. At the same time [^18^F]FDG-PET detected one additional patient with suspected liver metastases, which turned out to be unspecific tracer-uptake in the follow-up imaging. Intermodality comparison of lesion-to-background ratios in terms of [SUV_max_lesion_/SUV_mean_background_] for individual timepoints *t*_*1*_ and *t*_*2*_ revealed significant differences between [^18^F]FDG and [^68^Ga]FAPI (Wilcoxon-test at timepoint *t*_*1*_, *p* = 0.008; at timepoint *t*_*2*_, *p* = 0.005). At timepoint *t*_*1*_, mean [^68^Ga]FAPI lesion-to-background ratio was 11.2 (median 8.5, IQR 7.5–15.2, range 5.7–21.8) and thus, significantly higher than mean [^18^F]FDG lesion-to-background ratio of 4.4 (median 4.1, IQR 3.1–5.8, range 1.9–7.8). The same relation becomes evident for timepoint *t*_*2*_, where mean [^68^Ga]FAPI lesion-to-background ratio was 17.1 (median 12.3, IQR 9.7–25.6, range 6.7–35.9) and significantly higher than mean [^18^F]FDG lesion-to-background ratio of 2.5 (median 2.6, IQR 1.6–3.3, range 0.6–3.8). Comparing the three tumor entities no difference was found considering [^68^Ga]FAPI lesion-to-background ratios (*p* = 0.970, Kruskal Wallis test) or considering [^18^F]FDG lesion-to-background ratios (*p* = 0.741, Kruskal Wallis test) in this limited size series.

Additionally, to consider the background activity concentration in the segmentation of the MTV_*Background*_ the respective threshold was calculated as described by^27^. According to this formula at timepoint *t*_*1*_ mean [^68^Ga]FAPI-MTV_*Background*_ over background was 7.5 (median 7.2, IQR 5.5–8.4, range 4.8–12.0) and thus, likewise, significantly higher than mean [^18^F]FDG-MTV_*Background*_ over background of 5.2 (median 4.6, IQR 4.2–6.5, range 4.2–6.6) (Wilcoxon-test at timepoint *t*_*1*_, *p* = 0.038). Intermodality comparison of radiation therapy plans showed that [^68^Ga]FAPI-based planning resulted in a [^18^F]FDG MTV target volume dose coverage, characterized by mean D100% values of 97.2% and mean V95% values of 98.8% for [^18^F]FDG MTV. While [^18^F]FDG-based planning resulted in mean D100% of 35.9% and V95% of 78.1% for [^68^Ga]FAPI MTV. The D100% or V95% using [^68^Ga]FAPI-based planning were significantly higher than the respective values using [^18^F]FDG-based planning and reading out the coverage of the MTV (*p* < 0.012 and *p* < 0.018, Wilcoxon test). [^18^F]FDG-based planning resulted only in 2 patients in V95% above 95% for [^68^Ga]FAPI MTV, and in 1 patients in D100% above 97% for [^68^Ga]FAPI MTV. Plain morphologically delineated GTV coverage was 76.4% by the [^18^F]FDG-based planning and 99.5% by the [^68^Ga]FAPI-based planning in terms of V95%.

Altogether 10/14 patients underwent a second [^68^Ga]FAPI-PET/CT and 11/14 patients a second [^18^F]FDG-PET/CT at timepoint *t*_*2*_ with mean 50.3 days (range 26–78) from end of radiation therapy to 2nd [^68^Ga]FAPI-follow-up imaging considering those undergoing radiation therapy and 374 days considering the one patient receiving 2nd [^68^Ga]FAPI-follow-up imaging from 1st [^68^Ga]FAPI-PET/CT without radiation therapy. 2 patients underwent a third [^68^Ga]FAPI-PET/CT and 3 patients a third [^18^F]FDG-PET/CT at timepoint *t*_*3*_ (mean 111 days, range 103–119 from end of radiation therapy to 3rd [^68^Ga]FAPI-follow-up imaging).

**Figure 5:**
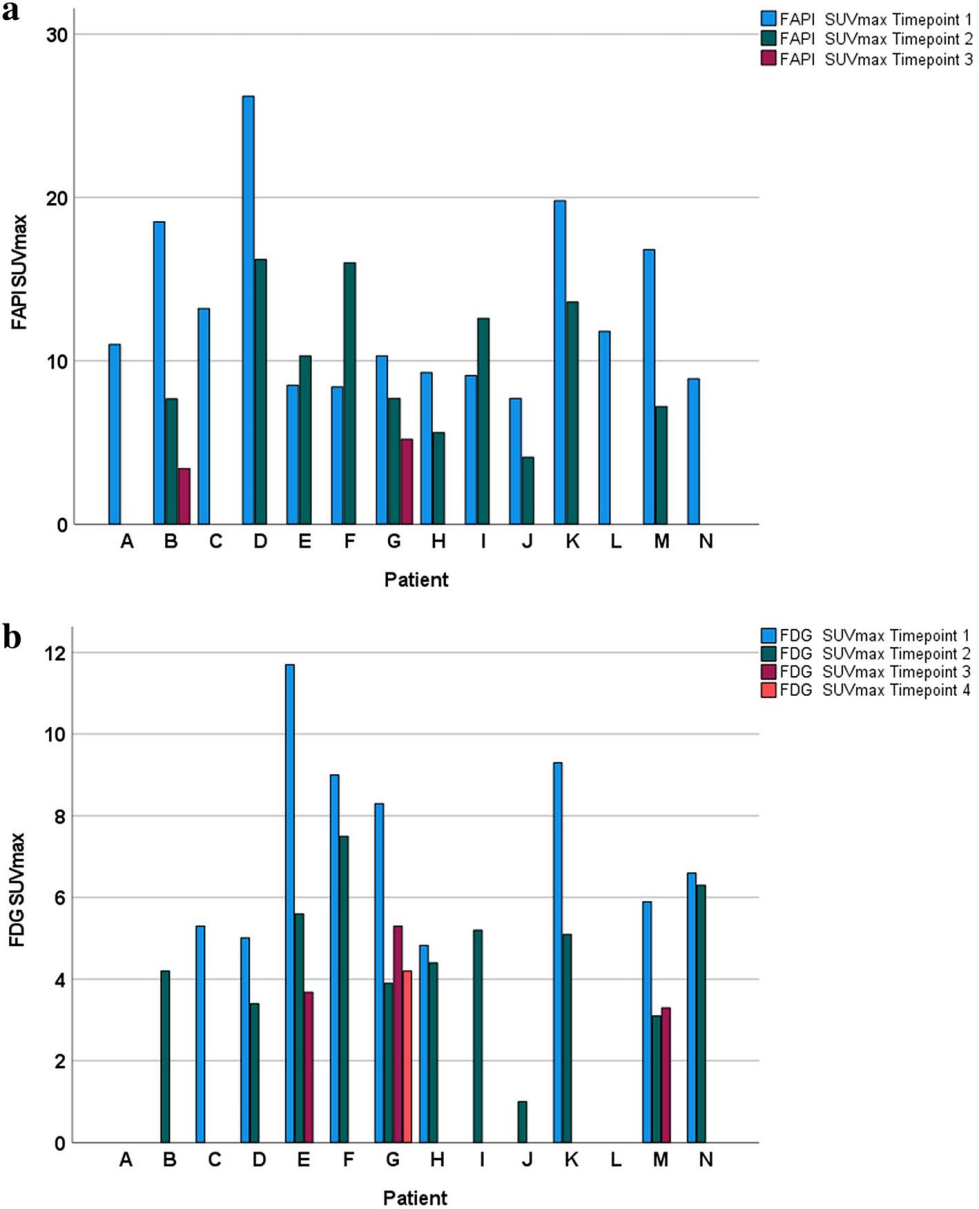
(**a**) Bar graph displaying change of [^68^Ga]FAPI SUV_max_ at timepoints *t*_1_, *t*_2_ and *t*_3_ for patient A–N (prior and after radiation therapy treatment completion), with patients A, C and I receiving no radiation therapy. Patient F developed fulminant peritoneal carcinomatosis evident on follow-up PET/CT. Patient A, C, L and N did not receive a second [^68^Ga]FAPI-PET/CT, while patient B and G underwent a third [^68^Ga]FAPI-PET/CT [adenoidcystic carcinoma A–C, biliary tract carcinoma D–G, pancreatic ductal adenocarcinoma (PDAC) H–N]. (**b**) Bar graph displaying change of [^18^F]FDG SUV_max_ at timepoints *t*_*1*_*, t*_*2*_ and *t*_*3*_ for patient A–N (prior and after radiation therapy treatment completion), with patients A, C and I receiving no radiation therapy. Patient A, B, I, J and L did not receive a [^18^F]FDG-PET/CT at timepoint *t*_*1*_, patients A, C and L did not receive a second [^18^F]FDG-PET/CT, while patient E, G and M underwent a third, and patient G a fourth [^18^F]FDG-PET/CT [adenoidcystic carcinoma A-C, biliary tract carcinoma D–G, pancreatic ductal adenocarcinomas (PDAC) H–N].

In 7/9 patients who received radiotherapy SUV_max_ of the index lesion declined on follow-up [^68^Ga]FAPI-PET/CT-imaging for more than 40% (mean decline 57.6%, range 41.4–74.8%), PERCIST-criteria suggest 30% cut-off. In 2/9 patients who received radiotherapy and a follow-up [^68^Ga]FAPI-PET/CT SUV_max_ increased for more than 20% (Fig. 5a). In both, there was a mismatch of SUV_max_ uptake between [^68^Ga]FAPI- and [^18^F]FDG-PET/CT. In one of these two patients (patient E) the size of the index lesion was regressive and central necrotic at follow-up of 6 months on MRI, on second and third follow-up [^18^F]FDG-PET/CT, which is considered as treatment response. Thus, in this case [^68^Ga]FAPI-increase did not indicate tumor progression, but a fibroblastic tissue response after ablative radiation therapy up to 50 Gy at 2.5 Gy/F. On the other hand, the other of the two patients (patient F), with [^68^Ga]FAPI-uptake increase for more than 90% at timepoint *t*_*2*_, developed a fulminant peritoneal carcinomatosis. Likewise, analyzing treatment response in those patients who underwent radiation therapy showed that [^68^Ga]FAPI MTV decreased from timepoint *t*_*1*_ to *t*_*2*_ to *t*_*3*_ in most patients (7/9), except of the afore mentioned 2. Analyzing treatment response with [^18^F]FDG revealed that [^18^F]FDG-SUV_max_ and MTV also decayed from *t*_*1*_ to *t*_*2*_ to *t*_*3*_. In all patients (n = 7) who received a pre- and posttreatment [^18^F]FDG-PET/CT as well as radiotherapy, SUV_max_ of the index lesion decreased by a mean of 33% on follow-up [^18^F]FDG-PET/CT-imaging (Fig. 5b).

Additionally, to further examine the impact of the perilesional background on [^68^Ga]FAPI uptake, the lesion-to-background difference was assessed in terms of %ΔSUV_maxTB_, the difference values of [SUV_*maxTumor*_ − SUV_*maxBackground*_] at timepoints *t*_*2*_ following *t*_*1*_ after treatment. With baseline SUV_*maxTumor*_ at timepoint *t*_*1*_ being 100%, the lesion-to-background difference %ΔSUV_maxTB_ increased for 14.5% in the respective patient F who developed a peritoneal carcinomatosis. In the only patient (patient I) with follow-up imaging who received no radiotherapy, SUV_max_ increased for 38.5% and lesion-to-background ratios remained stable on follow-up [^68^Ga]FAPI-PET/CT (Fig. 5a).

**Figure 6:**
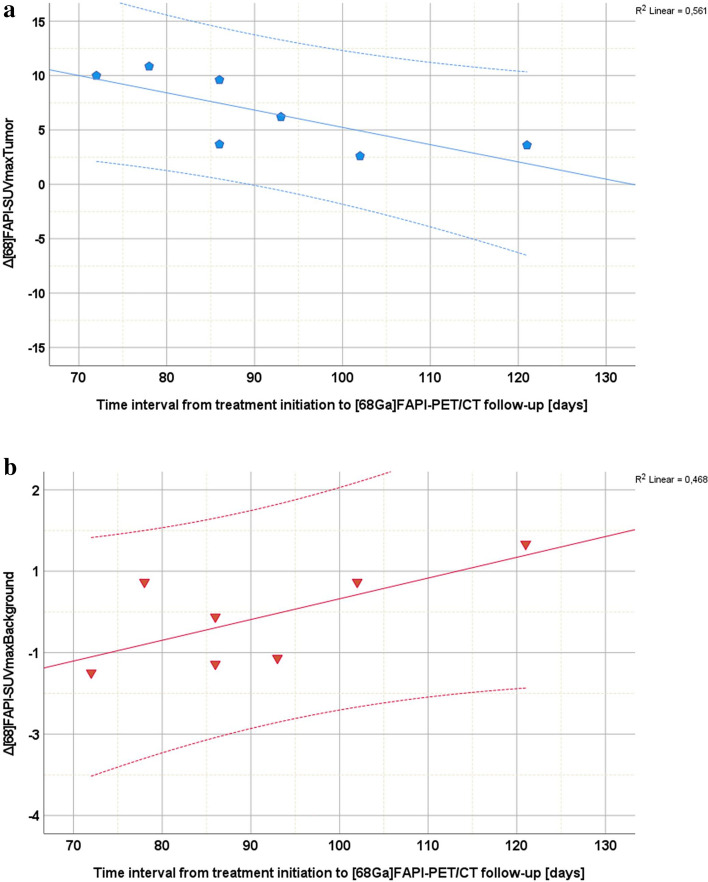
(**a**) Distribution plot highlighting absolute difference of [^68^Ga]FAPI-uptake for index lesions (blue spheres) (ΔSUV_maxTumor_) examined on timepoint *t*_*1*_ and timepoint *t*_*2*_. Absolute difference of tumoral uptake on follow-up PET-images declines. Scatter graphs considering the 7 patients undergoing radiation therapy with SUV_max_ decrease of index lesions from timepoint *t*_*1*_ to timepoint *t*_*2*_. (**b**) Distribution plot highlighting absolute difference of [^68^Ga]FAPI-uptake for perilesional background (red triangles) (ΔSUV_maxBackground_) examined on timepoint *t*_*1*_ and timepoint *t*_*2*_. Absolute difference of perilesional uptake on follow-up PET-images rises. Scatter graphs considering the 7 patients undergoing radiation therapy with SUV_max_ decrease of index lesions from timepoint *t*_*1*_ to timepoint *t*_*2*_.

In all patients receiving follow-up imaging the mean change of tumoral [^68^Ga]FAPI-uptake from timepoint *t*_*1*_ to *t*_*2*_ was − 14.7% (median − 34.7%, IQR − 49.4% to + 25.5%). Mean change of background [^68^Ga]FAPI-uptake from timepoint *t*_*1*_ to *t*_*2*_ was + 31.3% (median + 39.7%, IQR − 4.4% to + 54.0%). Mean change of tumoral [^18^F]FDG-uptake from timepoint *t*_*1*_ to *t*_*2*_ was − 32.5% (median − 38.7%, IQR − 51.0% to + 10.8%). Mean change of background [^18^F]FDG-uptake from timepoint *t*_*1*_ to *t*_*2*_ was − 3.7% (median − 11.4%, IQR − 27.0% to + 22.8%). To test for consistent differences between the percentage change of [^68^Ga]FAPI- and [^18^F]FDG-uptake from timepoint *t*_*1*_ to *t*_*2*_ the sign-test was used. While tumoral percentage change showed no significant difference between [^68^Ga]FAPI- and [^18^F]FDG-images (*p* = 1.0, two-sided Exact sign-test), background percentage change from *t*_1_ to *t*_2_ was significantly different between [^68^Ga]FAPI- and [^18^F]FDG-images (*p* = 0.016, two-sided Exact sign-test). This relation remained the same, when excluding the patient who underwent follow-up, but did not receive radiation therapy (tumoral percentage change *p* = 1.0, two-sided Exact sign-test; background percentage change *p* = 0.016, two-sided Exact sign-test). In patients with treatment response there was an apparent relation in tumor and background reaction, whereas absolute perilesional [^68^Ga]FAPI-uptake difference increased (*p* = 0.053, two-sided linear regression analysis), absolute tumoral [^68^Ga]FAPI-uptake difference decreased from radiation therapy start to follow-up [^68^Ga]FAPI-imaging (*p* = 0.09, two-sided linear regression analysis) (timepoint *t*_1_ to *t*_2_, Fig. 6a,b) indicating a time trend (Fig. 6a,b).

Interestingly, SUV_max_ (Wilcoxon-test, *p* = 0.037) and SUV_mean_ (Wilcoxon-test, *p* = 0.012) of perilesional background on [^68^Ga]FAPI-PET/CT-images significantly increased for all patients receiving follow-up [^68^Ga]FAPI-PET/CT from timepoint *t*_*1*_ to *t*_*2*_, except for one, with mean values depicted in Table 2. Meanwhile SUV_max_ (Wilcoxon-test, *p* = 0.499) and SUV_mean_ (Wilcoxon-test, *p* = 0.670) of perilesional background on [^18^F]FDG-PET/CT-images remained stable or slightly decreased without significant changes from timepoint *t*_*1*_ to *t*_*2*_ (Table 2).

**Table 2:** Displaying mean and median change of SUV_mean_ and SUV_max_ of perilesional background on [^68^Ga]FAPI- on [^18^F]FDG-PET/CT-images at timepoints *t*_1_, *t*_2_ and *t*_3_ for patient A–N (prior and after radiation therapy treatment completion), with patients A, C and I receiving no radiation therapy. Patient A, C, L and N did not receive a second follow-up [^68^Ga]FAPI-PET/CT (n = 4), while patient B and G underwent a third [^68^Ga]FAPI-PET/CT (n = 2). Blue arrows indicating direction of uptake change on follow-up imaging at timepoint *t*_2_ in comparison to timepoint *t*_1_.

	[^68^Ga]FAPI						[^18^F]FDG					
	Timepoint *t*_*1*_		Timepoint *t*_*2*_		Timepoint *t*_*3*_		Timepoint *t*_*1*_		Timepoint *t*_*2*_		Timepoint *t*_*3*_	
N	SUV_mean_	SUV_max_	SUV_mean_	SUV_max_	SUV_mean_	SUV_max_	SUV_mean_	SUV_max_	SUV_mean_	SUV_max_	SUV_mean_	SUV_max_
Follow-up	14	14	10	10	2	2	9	9	11	11	3	3
No follow-up	0	0	4	4	12	12	5	5	3	3	11	11
Mean	1.2	3.9	1.6↑	5.0↑	1.3	4.3	1.8	3.8	1.8	3.6↓	1.9	3.4
Median	1.1	3.4	1.5↑	3.8↑	1.3	4.3	1.7	3.6	1.6↓	3.5↓	1.8	3.0
**Percentile**												
25	1.0	2.5	1.4↑	3.4↑	0.8	4.1	1.4	3.1	1.5	3.0↓	1.3	2.7
50	1.1	3.4	1.5↑	3.8↑	1.3	4.3	1.7	3.6	1.6↓	3.5↓	1.8	3.0
75	1.3	4.9	1.7↑	5.4↑			2.3	4.6	2.3	4.2↓		

## Discussion

The primary aim of the present study was to examine the impact of pretreatment [^68^Ga]FAPI-PET/CT on target volume definition for patients with biliary tract, pancreatic ductal adenocarcinomas (PDAC) and adenoidcystic malignancies presented to radiation therapy. Our results reveal that [^68^Ga]FAPI-based planning was feasible covering both, plain macroscopic gross tumor volume and [^18^F]FDG-volume, while [^18^F]FDG-based planning did not entirely cover [^68^Ga]FAPI-volume. Lesions were considerably larger and better contrasted with higher lesion-to-background ratios within [^68^Ga]FAPI-images compared to [^18^F]FDG-images. But larger MTVs do not automatically mean larger PTVs or higher toxicities^30^. In a multicentre, open-label, randomised, controlled [^18^F]FDG-PET/CT trial examining patients with locally advanced non-small-cell lung cancer, the median gross tumor volume was indeed higher in the [^18^F]FDG PET-based target group compared to the conventional target group; however, there was no significant difference in the median planning target volume (*p* = 0.180)^30^. If the detection is more sensitive, then the elective CTV volume, also concerning the lymph node stations, can potentially be reduced.

In line with previous studies^16^ we observed that [^68^Ga]FAPI provided high-contrast images. Particularly in tumor entities, which showed a low or absent [^18^F]FDG-uptake, [^68^Ga]FAPI enhanced target definition for radiation therapy planning. The present results confirm findings of preceding studies identifying [^68^Ga]FAPI as an important diagnostic tool for a range of cancer types^31,32^. A recent study on [^68^Ga]Ga-DOTA-FAPI-04 PET/CT showed a superior detection efficacy of [^68^Ga]Ga-DOTA-FAPI-04 PET/CT for detecting various primary and metastatic lesions compared to [^18^F]FDG PET/CT^32^. Significantly higher SUV_max_ uptake values are reported for stomach, liver and pancreatic duct adenocarcinoma compared to [^18^F]FDG PET/CT^32^.

Contrary to [^18^F]FDG which detects pathologies with enhanced glycolysis, [^68^Ga]FAPI uses other cancer and specific cell-surface targets. It binds to tumor associated fibroblasts rather than to normal adjacent tissue, where it is rapidly degraded. As FAP is a transmembrane glycoprotein on cancer-associated fibroblasts (CAFs) of several tumor entities^13^, it is probable that radiation treatment planning for a wider range of cancer types may profit^31,33^. Furthermore, some authors claim that the high, rather selective uptake and excellent image contrast on [^68^Ga]FAPI PET/CT may allow new applications for noninvasive lesion characterization, staging examinations, or radioligand therapies^26^. Likewise, Chen et al. report in a study of 59 cancer patients, that 22 patients presented with inconclusive [^18^F]FDG-PET findings^34^, while 86.4% (19/22) of these inconclusive lesions, primarily from liver cancer (6/19, 31.6%) and gastric cancer (5/19, 26.3%), showed high [^68^Ga]FAPI-uptake^34^. However, other authors argument that [^68^Ga]FAPI is not more tumor-specific than [^18^F]FDG, but that it is more sensitive to certain kinds of inflammations, like pancreatic lesions with characteristic storiform fibrosis and IgG4-related disease^35^. It must not be neglected that [^68^Ga]FAPI as other radiopharmacons, may be prone to unspecific tracer uptake^36^. It is reported that degenerative lesions, muscle, head-and-neck, scarring, mammary glands or uterus may show an increased tracer uptake^36^. According to Chen et al. in the lesion-based analysis sensitivity, specificity, positive predictive value, and negative predictive value of [^68^Ga]-DOTA-FAPI-04 PET/CT for the diagnosis of suspicious lesions, which were inconclusive on [^18^F]FDG-PET, were 94.5%, 58.2%, 84.0%, and 82.1%, respectively^34^. The false-positive findings were attributed to inflammatory-induced fibrosis^34^. Particularly posttreatment scarring and fibrotic tissues after surgery may represent a challenge for radiation therapy planning. Thus, precise knowledge of surgical operations and maneuvers remains crucial for optimal treatment planning, which may not be replaced by mere PET-imaging demanding morphological information from the CT- or MRI-component.

**Figure 7:**
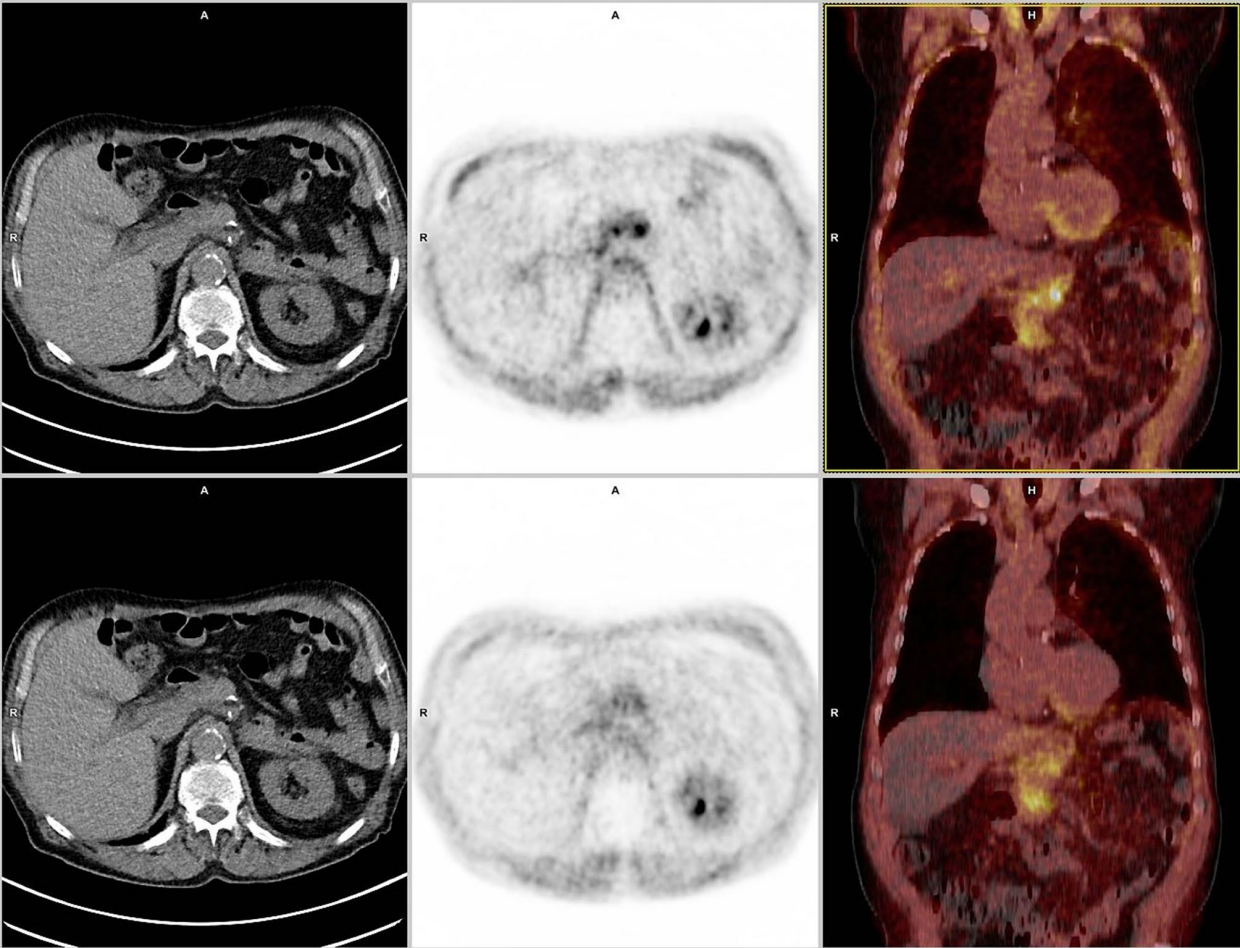
Showing patient J with a recurrent pancreatic ductal adenocarcinoma at timepoint *t*_*1*_ (upper row) (timepoint *t*_*1*_ SUV_max_ 8 and MTV 63 cm^3^) and posttreatment images at timepoint *t*_*2*_ (lower row) (78 days after radiation therapy treatment completion; timepoint *t*_*2*_ SUV_max_ 4 and MTV 29 cm^3^). Upper and lower left-side: unenhanced CT; upper middle column: pretreatment [^68^Ga]FAPI-PET; lower middle column: posttreatment [^68^Ga]FAPI-PET (both axial reconstructions at the level of the liver and left kidney); upper and lower right-side: fused mode [^68^Ga]FAPI-PET/CT at timepoint *t*_*1*_ (upper row) and [^68^Ga]FAPI-PET/CT at timepoint *t*_*2*_ (lower row) [point-spread function (PSF) reconstruction; PET Heat 2D, 13 min after 52 MBq [^68^Ga]FAPI-injection at timepoint *t*_*1*_ and 16 min after 95 MBq [^68^Ga]FAPI-injection at timepoint *t*_*2*_]. Focal pathological uptake on follow-up PET-images considerably declined.

A second aim of the present study was the evaluation of the post-radiation treatment [^68^Ga]FAPI-PET/CT for response assessment. First in 1999 the European Organization for Research and Treatment of Cancer (EORTC) recommended response criteria for solid tumors based on PET-imaging^37^. These criteria are dedicated to [^18^F]FDG, but the translation to other radiopharmacons seems reasonable. A complete metabolic response on [^18^F]FDG is defined in cases, where tumor lesions are no longer detectable in contrast to the background uptake. Progressive metabolic disease is defined as an increase in SUV_max_ of ≥ 25% compared to the pretreatment PET-imaging, while a partial metabolic response is defined as SUV_max_-decline for 15–25% or more than 25% after one or more cycles of chemotherapy^31^. Moreover, the EORTC first proposed PET/CT for treatment response assessment after immunotherapy^38^. PERCIST 1.0 criteria turned out to be important in evaluating response to novel cancer therapies that stabilize disease^39^. Additionally, volumetric parameters reported on interim [^18^F]FDG-PET/CT-imaging are identified as valid prognostic factors^5–7^. Though, as discussed above it is known that both, [^68^Ga]FAPI- and [^18^F]FDG-imaging may result in false positive and false negative results, in the present study combined posttreatment tumoral [^68^Ga]FAPI- and [^18^F]FDG-uptake after treatment completion showed a continuous decrease in SUV_max_ in all but 2 patients after radiation therapy helping to determine treatment response (Fig. 7). In the two patients there was a mismatch of SUV_max_ uptake between [^68^Ga]FAPI- and [^18^F]FDG-PET/CT. One of these two patients had a treatment response with regressive and central necrotic index lesion at follow-up of 6 months, thus, in this case [^68^Ga]FAPI-increase indicates a fibroblastic tissue response after ablative radiation therapy up to 50 Gy at 2.5 Gy/F. On the other hand, the other patient with [^68^Ga]FAPI-uptake increase for more than 90% at timepoint *t*_*2*_, developed a fulminant peritoneal carcinomatosis. In the one patient who received no radiation therapy and who underwent [^68^Ga]FAPI-follow-up imaging, SUV_max_ of the index lesion increased for more than 38%. When assessing treatment response with [^68^Ga]FAPI-imaging, it is crucial to discuss perilesional background reaction. Our results indicate that posttreatment the tumoral [^68^Ga]FAPI-uptake and perilesional background [^68^Ga]FAPI-uptake differ, which harbours a fibroblastic radiation induced stroma response. These results are supported by our observation that absolute perilesional [^68^Ga]FAPI-uptake difference increased in dependence of time from treatment initiation to follow-up. Previous studies discussed that irradiation of murine subcutaneous stroma leads to retarded tumor growth, an effect called Tumor Bed Effect (TBE)^40^. TBE is known to impact the sensitivity of stromal tissue to radiation^40^. While perilesional and tumoral [^18^F]FDG uptake behaved uniformly, perilesional and tumoral reaction may differ in [^68^Ga]FAPI-imaging. Complementary [^68^Ga]FAPI- and [^18^F]FDG-imaging enhance treatment response assessment.

Finally, the question about radiation dose remains. Giesel et al. report an equivalent dose of ~ 3–4 mSv for a [^68^Ga]FAPI-examination similar to dose values published for [^18^F]FDG-, [^68^Ga]DOTATATE, and [^68^Ga]PSMA-11-examinations^16^.

## Conclusion

[^68^Ga]FAPI-PET helped to delineate active primary or recurrent tumor lesions in three tumor entities. Non-tumor specific [^68^Ga]FAPI-uptake for e.g. degenerative lesions, cicatricial tissue, muscles or in the head-and-neck region have to be considered. The [^18^F]FDG-based planning did not cover the [^68^Ga]FAPI-volume in the majority of cases, whereas the [^18^F]FDG-volume was covered by the [^68^Ga]FAPI-based planning. Pre- and post-treatment [^68^Ga]FAPI-PET/CT improved radiotherapy planning and treatment response assessment in this particular patient group.

## Supplementary Information

Supplementary Information.

## Data Availability

The datasets used and analysed during the current study available from the corresponding author on reasonable request.
