# Supplementary material for: [68Ga]FAPI-PET/CT for radiation therapy planning in biliary tract, pancreatic ductal adeno-, and adenoidcystic carcinomas
Source: Sci Rep. 2022 Sep 28;12:16261. doi: 10.1038/s41598-022-20447-6 (PMC9519639; doi:10.1038/s41598-022-20447-6)
Supplement: Supplementary file 1 — Supplementary Information. [file 41598_2022_20447_MOESM1_ESM.docx]

| **Patient ID** | | **Sex** | **Age** | **Entity** | **RTx** | **Relapse prior to radiotherapy at time of RTx-indication** | **Medical history** |
| --- | --- | --- | --- | --- | --- | --- | --- |
| **A** | m | | 60 | Adenoidcystic carcinoma | 0 | 1 | Secondary re-resection with sufficient surgical margins |
| **B** | f | | 69 | Adenoidcystic carcinoma | 1 | 0 | Primary inoperable |
| **C** | f | | 55 | Adenoidcystic carcinoma | 0 | 0 | Radiation therapy at a different site with carbon ions instead of photons |
| **D** | m | | 39 | BTC | 1 | 1 | Intrahepatic relapse after initial resection |
| **E** | f | | 54 | BTC | 1 | 1 | Lymphogenic recurrence after initial hemihepatectomy right |
| **F** | f | | 54 | BTC | 1 | 1 | Tumor recurrence after pylorus-preserving pancreatic head and extrahepatic BTC-resection |
| **G** | m | | 45 | BTC | 1 | 1 | Early relapse after hemihepatectomy left |
| **H** | f | | 57 | PDAC | 1 | 1 | Lymphogenic recurrence |
| **I** | f | | 60 | PDAC | 0 | 1 | RTx cancelled due to newly diagnosed peritoneal carcinomatosis |
| **J** | m | | 76 | PDAC | 1 | 1 | Initial tumor resection in toto with early relapse |
| **K** | m | | 74 | PDAC | 1 | 0 | Primary inoperable |
| **L** | m | | 55 | PDAC | 1 | 1 | Lymphogenic recurrence after resection |
| **M** | f | | 67 | PDAC | 1 | 1 | Early lymphogenic recurrence after resection |
| **N** | m | | 65 | PDAC | 1 | 1 | Tumor recurrence after left-sided pancreatic resection |

| **Supplement Table 1** | Patient characteristics of all consecutive patients with adenoidcystic carcinoma, cholanangiocellular/ biliary tract (BTC), and pancreatic ductal adenocarcinomas (PDAC) receiving [^68^Ga]FAPI-PET/CT for radiation therapy planning (sex, age, tumour entity; RTx: conducted: RTx 1 yes, 0 no; relapse at time of medical RTx-indication prior to radiotherapy: 1 yes, 0 no; medical history [adenoidcystic carcinoma A-C, biliary tract carcinoma D-G, PDAC H-N]. |
| --- | --- |
